# Unraveling the Early Life Impacts on Healthy Aging: Insights From the China Health and Retirement Longitudinal Study

**DOI:** 10.1093/geroni/igaf122.3019

**Published:** 2025-12-31

**Authors:** Liangyi Jin, Chenkai Wu

**Affiliations:** Duke Kunshan University, Kunshan, Jiangsu, China (People’s Republic); Duke Kunshan University, Kunshan, Jiangsu, China (People’s Republic)

## Abstract

Early-life socioeconomic and environmental factors play a crucial role in influencing the onset of diseases in later life. However, their impact on healthy aging remains understudied. This study examined the association between early-life circumstances and healthy aging among community-dwelling older adults in China, assessed by the Chinese Healthy Aging Index (CHAI). Data were from the China Health and Retirement Longitudinal Study, including 3,069 participants aged ≥60 years who completed the life history survey and had data to calculate the CHAI. The CHAI comprised six components, and each was scored 0 (healthiest), 1, and 2 (unhealthiest) according to sex-specific tertiles or clinically relevant cut-points. The scores were summed to construct the CHAI (0–12). Seven Early life risk factors were included, and we used the logistic regression to determine their associations with CHAI both individually and cumulatively. The average CHAI score was 5.91 (SD = 2.10); 4.82% had a score of 0-2 (healthiest). After multivariable adjustment, personal education attainment and family economic status were individually associated with healthier CHAI scores; the cumulative early-life risk factor score was significantly associated with the CHAI, both in categories and continuously. Each additional score was related to a 34% (95% CI: 20%, 50%) higher odds of being in the healthiest CHAI category. Favorable early-life socioeconomic conditions are associated with healthier aging outcomes among Chinese community-dwelling older adults. Addressing socioeconomic inequalities early in life is pivotal in promoting healthy aging.

